# Key Features of Gamma-Delta T-Cell Subsets in Human Diseases and Their Immunotherapeutic Implications

**DOI:** 10.3389/fimmu.2017.00761

**Published:** 2017-06-30

**Authors:** Myriam Lawand, Julie Déchanet-Merville, Marie-Caroline Dieu-Nosjean

**Affiliations:** ^1^Cordeliers Research Center, UMRS 1138, Team “Cancer, Immune Control and Escape”, INSERM, Paris, France; ^2^Cordeliers Research Center, UMRS 1138, University Sorbonne-Paris Cité, University Paris Descartes, Paris, France; ^3^Cordeliers Research Center, UMRS 1138, University Pierre and Marie Curie (UPMC), Paris 06, University Paris-Sorbonne, Paris, France; ^4^ImmunoConcEpT, CNRS UMR 5164, University of Bordeaux, Bordeaux, France

**Keywords:** human gamma-delta T cell, cytotoxicity, cytokine, NK receptor, antigen presentation, cancer, infection, autoimmunity

## Abstract

The unique features of gamma-delta (γδ) T cells, related to their antigen recognition capacity, their tissue tropism, and their cytotoxic function, make these cells ideal candidates that could be targeted to induce durable immunity in the context of different pathologies. In this review, we focus on the main characteristics of human γδ T-cell subsets in diseases and the key mechanisms that could be explored to target these cells.

## Introduction

Gamma-delta (γδ) T cells are an important subset of “unconventional” T lymphocytes as they have the ability to recognize a broad range of antigens without the presence of major histocompatibility complex (MHC) molecules. They can attack target cells directly through their cytotoxic activity or indirectly through the activation of other immune cells. γδ T-cell functional responses are induced upon the recognition of stress antigens, which promotes cytokine production and regulates pathogen clearance, inflammation, and tissue homeostasis in response to stress (1).

However, given that different parameters concerning human γδ T-cell function and phenotype within tissues are still not well understood, it is important to be updated on the current state of knowledge of human γδ T cells. This will permit a better understanding of what could be performed in the future and how to target them in order to improve the management of patients.

For this aim, this review describes the main features of human γδ T-cell subsets in various pathologies and discusses the mechanisms by which they influence the outcome of the immune response that could be targeted for immunotherapy.

## Pleiotropic Role of Human γδ T Cell Subsets

In humans, there are two major subsets of γδ T cells identified by their Vδ chain. Vδ1 T cells are predominant in the thymus and peripheral tissues and recognize various stress-related antigens mostly uncharacterized. Vδ2 T cells constitute the majority of blood γδ T cells (2). They always associate to the Vγ9 chain in adults and mainly recognize phosphoantigens, i.e., phosphorylated non-peptidic molecules that are metabolic intermediates of the isoprenoid biosynthesis (3).

### Major Cytokines and Cytotoxic Potential

Both human γδ T-cell subsets exhibit a cytotoxic potential that is induced through the expression of cell surface receptors [i.e., γδ TCR (T-cell receptor) and NKG2D (natural killer group 2D)] and is preponderantly mediated by the release of soluble mediators (i.e., perforin and granzymes) (4, 5). They can produce granulysin, which is a potent anti-microbial protein (6, 7), and express ligands such as CD95L and Tumor necrosis factor-related apoptosis-inducing ligand, which engage several death receptors on target cells. In addition, they can kill their targets indirectly through antibody-dependent cellular cytotoxicity (ADCC) in a CD16-dependent mechanism (8). Other molecules such as DNAM-1 (DNAX accessory molecule-1), leukocyte function-associated antigen-1, and the co-stimulatory receptor CD27 are also involved in γδ T-cell activation and cytotoxicity (9).

Importantly, cord blood naïve γδ T cells can differentiate into the IL-17^+^IFN-γ^−^ Vγ9Vδ2 T cells with a cytotoxic potential in the presence of IL-23 and a TCR signaling (10). In contrast, thymic naïve γδ T cells secrete IFN-γ in the presence of IL-2 or IL-15, through the *de novo* expression of T-bet and eomesodermin, and the release of cytotoxic molecules against leukemia cells (11). Other studies reported IL-17^+^ γδ T-lymphocyte differentiation in the presence of IL-7 (12) or other activation stimuli (13) and high inflammatory conditions (14, 15).

Altogether, human γδ T cells represent key actors of the immunity because of their pro-inflammatory phenotype and cytotoxic potential.

### Antigen Presentation Capacity

Human γδ T cells can exhibit an antigen-presenting capacity. Similar to dendritic cells (DCs), blood Vγ9Vδ2 T cells are able to respond to signals from microbes and tumors and prime CD4^+^ and CD8^+^ T cells (16). Indeed, γδ T-APCs were also described to cross-present antigens to CD8^+^ T cells (17). The intracellular protein degradation and endosomal acidification are significantly delayed in γδ T cells in comparison to monocyte-derived DCs (18). The antigens are transported across IRAP (Insulin-Regulated AminoPeptidase)-positive early and late endosomes (19), and their processing consists of an export to the cytosol for degradation by the proteasome before being imported into a MHC-I-loading compartment (18). Moreover, activated γδ T cells are able to phagocytose tumor antigens and apoptotic or live cancer cells possibly through the scavenger receptor CD36 in a C/EBPα (CCAAT/enhancer-binding protein α)-dependent mechanism and mount a tumor antigen-specific CD8^+^ T-cell response (20). Moreover, γδ T cells can induce DC maturation through TNF-α production (21, 22).

Overall, γδ T cells can process a wide range of antigens for presentation and stimulate other immune cells. Therefore, their implication in response to infections or cancer would help to design new strategies in order to improve clinical response of human γδ T cell-based immunotherapy.

### Key Receptors in Immune Surveillance

Different receptors namely the TCR, co-stimulatory molecules, and NK receptors play a key role in the regulation of γδ T-cell-mediated immune responses [reviewed in Ref. (23)]. For instance, the activation of blood Vγ9Vδ2 T cells by anti-NKG2D antibody or its ligand MICA (MHC class I chain-related sequence A) induces TNF-α production and the release of cytolytic granules (24). Moreover, the triggering of NKG2D enhanced their response to microbe-associated antigens (25). In lymphocytic leukemia, a hematologic tumor highly resistant to activated Vγ9Vδ2 T cells, IL-2 or IL-15, and TCR stimulation upregulates the expression of NK receptors NKp44, NKp46, and NKp30 on Vδ1^+^ T cells, allowing their acquisition of cytotoxicity against leukemia cells (26). DNAM-1 engagement can also promote the activation of Vδ2 T cells and ultimately, the killing of tumor cells (27, 28). Phosphoantigen stimulation of Vγ9Vδ2 T cells is able to induce TNF-α production through the upregulation of CD16 expression (29). Its role in mediating ADCC was highlighted using therapeutic antibodies such as anti-CD20 (Rituximab) (30) and anti-HER2 antibody (Trastuzumab) (31).

The CD27–CD70 axis can enhance phosphoantigen-dependent activation, survival, proliferation, and secretion of pro-inflammatory cytokines of Vγ9Vδ2 T cells (32). These results suggest that CD27 can modulate Vδ2 T-cell activation and hence seems to be a major tool that could be manipulated in clinical settings. Of note, CD27 is expressed on Vδ1^+^ cells and, thus, may also play a role in their effector functions (32).

The promotion of a robust NK cell-mediated antitumor cytotoxicity has also been described through CD137 (4-1BB) engagement on blood activated γδ T lymphocytes which in turn induces the upregulation of NKG2D by NK cells, followed by the eradication of tumor cells (33).

In contrast, regulatory receptors for self-MHC class I molecules, particularly KIR (Killer cell Immunoglobulin-like Receptor) and LIR (Leukocyte Immunoglobulin-like Receptor) receptors, were reported to negatively regulate γδ T-cell activation (34, 35). This inhibition is due to the presence of intracytoplasmic ITIM (Immunoreceptor Tyrosine-based Inhibitory Motif) motif in the sequence of these receptors which turn off the activation signals upon phosphorylation. The ligation of BTLA (B- and T-Lymphocyte Attenuator), another regulatory receptor strongly expressed by resting Vγ9Vδ2 T cells, attenuates their own proliferation (36). The engagement of PD-1 (programmed cell death-1) expressed on activated γδ T cells downregulates IFN-γ production and their cytotoxic function (37).

Understanding the role of these mechanisms in γδ T cell-implication in pathological situations needs further investigations that would be important to develop proper strategies targeting these activation and inhibitory receptors. This would ensure an efficient activation of human γδ T cells in immune surveillance against tumors, pathogens, or autoimmunity and ultimately avoid undesired cytotoxicity against the host through a better discrimination between normal and altered tissues.

## Human γδ T-Cell Subsets in Cancer, Infectious Diseases, and Autoimmunity

### Pro- and Antitumor Effect of γδ T-Cell Subsets

Tumor-infiltrating γδ T cells have been demonstrated to be the most favorable prognostic immune population among many cancer types (38), in agreement with their capacity to kill different tumor cells like leukemia, neuroblastoma, and carcinomas (9).

Vδ1 and Vγ9Vδ2 T cells express distinct chemokine receptors (39, 40) and cell adhesion molecules (41) (Table 1), suggesting different homing mechanisms in tumors that can be selectively targeted for immunotherapy. Moreover, isolated Vδ1 lymphocytes from human lung tumors can selectively lyse autologous malignant cells *ex vivo* (42). Interestingly, administration of Vγ9Vδ2 T cells at suitable intervals after chemotherapy and zoledronate treatment increase the cytotoxic function and IFN-γ production by γδ T cells followed by a complete lysis of tumor cells in different malignancies (43). Different studies also reported the activation of γδ T cells after phosphoantigen or aminobiphosphonate injection, an approach which provides promising clinical activity by improving the cytotoxicity of γδ T cells particularly in presence of tumor-targeting antibody (44). In melanoma, tumor-infiltrating effector-memory γδ T cells have been shown to control tumor growth through distinct cytotoxic mechanisms (45) (Table 1). Furthermore, Vδ1 T cells were less susceptible to activation-induced cell death and could persist in the circulation for many years, which is in favor of a durable antitumor immunity (46) (Table 1).

**Table 1:** Main features of human γδ T-cell subsets in cancers.

Pathology	Human γδ T-cell subset features			Reference
	Vδ1^+^	Vδ2^+^	Other Vδ2^−^ (non-Vδ1)	
Solid cancers	Lysis of the autologous tumor when extracted from TILs and expanded *in vitro* in the presence of IL-2	–	–	(42)
	–	Cytotoxic function, IFN-γ production, and almost a complete lysis of tumor targets in different malignancies (after chemotherapy and zoledronate treatment)	–	(43)
		Expansion and improved cytotoxicity in the presence of phosphoantigens, amino-biphosphonates, or a tumor-targeting antibody in cancer immunotherapy		Reviewed in Ref. (44)
	CCR5 expression for migration to tumor sites	CCR5 and CXCR3 expression (consistent with a Th1-like phenotype)	–	(39)
	CCR2 expression for migration to tumor sites (antitumor effect: production of IFN-γ and cytotoxic function)	No CCR2 expression detected	–	(40)
	Expression of various adhesion molecules: LFA-1, VLA-α4 (CD49d), VLA-α5 (CD49e), L-selectin (CD62-L), and αEβ7(CD103)	Only LFA-1, L-selectin, and CD44v6 expression	–	(41)
	Ability to kill tumor cells of all melanoma-isolated Vδ1 cell lines	Significant cytotoxic activity for only two out of eight Vδ2 cell lines	–	(45)
	Lower susceptibility to activation-induced cell death, persistence in the circulation for many years (durable immunity)	–	–	Reviewed in Ref. (46)
	Major cellular source of IL–17 (pro-tumor role: chronic inflammation in CRC patients)	–	–	(15)
	Immunosuppressive and regulatory properties, such as suppression of dendritic cell maturation, T-cell proliferation, and IL-2 secretion	–	–	(47)
Hematological malignancies	–	Activation of Vγ9Vδ2 T cells by zoledronate: cytotoxicity largely dependent on granule exocytosis and partly on TRAIL pathways, TCR-mediated, and dependent on isoprenoid production by leukemia cells	–	(48)
	–	Leukemia/lymphoma cell killing by γδ T cells essentially mediated by ULBP1/NKG2D interaction	–	(49)
	Cytotoxicity against lymphoid leukemia cells associated with the expression of several NK receptors (mainly NKp30)	–	–	(26)
	–	–	Increased percentage of Vδ2^−^ T cells following CMV infection in kidney transplant recipients associated with reduced cancer risk (among which lymphoma)	(50)

*CMV, cytomegalovirus; CRC, colorectal cancer; LFA-1, leukocyte function-associated antigen-1; NKG2D, natural killer group 2D; TIL, tumor-infiltrating lymphocyte; TRAIL, tumor necrosis factor-related apoptosis-inducing ligand; ULBP1, UL16-binding protein 1; VLA-α4, very late antigen-4*.

Lamb et al. reported an association between the increased frequency of γδ T cells and improved disease-free survival of leukemia patients who received αβ T cell-depleted bone marrow transplants (51). An increased percentage of Vδ2^−^ T cells following cytomegalovirus (CMV) infection in kidney transplant recipients was also associated with reduced cancer risk (i.e., lymphoma) (50), suggesting a protective role of γδ T cells. In the context of chronic myelogenous leukemia (CML), the activation of Vγ9Vδ2 T cells by zoledronate was shown to increase anti-leukemia activities in a NKG2D-dependent manner, notably in CML-resistant patients (48). Indeed, UL16-binding protein 1 (ULBP1) is involved in lymphoma susceptibility to γδ T-cell-mediated cytolysis and the blockade of its receptor NKG2D significantly inhibits lymphoma cell killing. Thus, the authors propose that γδ T cytotoxic function is achieved through a two-step process (a TCR stimulation presumably by endogenous phophoantigens and a tumor cell recognition by NKG2D) and that ULBP1 could be used as a leukemia/lymphoma biomarker in clinical trials (49). Moreover, since Vδ1^+^ T cells were shown to express several NK receptors that are correlated with a high cytotoxic potential (26), they may constitute a potent therapeutic lymphocyte population that could be targeted for the immunotherapy of lymphocytic leukemia patients that are resistant to activated Vγ9Vδ2 T cells.

Nonetheless, a pro-tumor role of Vδ1 T cells has also been reported. Indeed, in colorectal cancer (CRC), these cells are responsible of the chronic inflammation in CRC patients through IL-17 secretion (15). In addition, some Vδ1 populations could exhibit immunosuppressive properties (i.e., inhibition of DC maturation, suppression of T-cell proliferation and IL-2 secretion), a function that can also be exploited for cancer therapy (47) (Table 1). A high γδ T-cell infiltrate in breast tumors was positively correlated with advanced tumor stages, HER2 expression status, and lymph node metastasis and ultimately associated with poor outcome of the patients (52). In this study, γδ T cells were considered as the most significant independent prognostic factor among many parameters including clinical grade and, thus, may serve as a valuable biomarker and potential therapeutic target for breast cancer.

Altogether, the natural contribution in tumor immunosurveillance and the effector functions of Vγ9Vδ2 T cells represent major advantages that have to be better exploited alone or in combination with current therapies (i.e., phosphoantigens + monoclonal antibodies). However, repeated injection of phosphoantigens may also lead to the anergy or exhaustion of effector γδ T cells. From an immunotherapeutic standpoint, the rather limited antitumor efficacy of adoptively transferred Vδ2 T cells and active immunotherapy trials using Vδ2 agonists can be the result of a suboptimal recognition of *ex vivo* tumor cells, presumably due to insufficient phosphoantigen levels. Therefore, it is now critical to better characterize human γδ T-cell subsets and the engaged mechanisms in individual cancers, especially the stage of differentiation, the activation status, and the immune checkpoint (ICP)/ICP-ligand expression, to irreversibly convert them toward an antitumor function for efficient immunotherapy.

### γδ T-Cell Subsets in Infections

γδ T-cell subsets have been described as potent effector populations against pathogens (Table 2). Indeed, Vγ9Vδ2 T cells can recognize phosphoantigens that are overexpressed in the methyl-erythritol phosphate biochemical pathway (MEP, also called DOXP (1-desoxy-d-xylulose-5-phosphate) pathway), a pathway which is used by many bacteria, fungi and parasites, such as *Plasmodium falciparum, Mycobacterium tuberculosis, Toxoplasma gondii*, and *Listeria monocytogenes* [reviewed in Ref. (53, 54)]. Vγ9Vδ2 T cells are thus activated and able to expand in the blood of infected individuals.

**Table 2:** Main features of human γδ T-cell subsets in infectious diseases.

Pathology	Human γδ T-cell subset features			Reference
	Vδ1^+^	Vδ2^+^	Other Vδ2^−^ (non-Vδ1)	
CMV infection	–	–	Reactivity of different Vδ2^−^ γδ T cell clones against CMV-infected cells	(55)
	–	–	Recognition of CMV-targeted endothelial cells and epithelial tumors (Vγ4Vδ5 clone interaction with EPCR)	(56)
	Antibody response and reactivity against CMV-infected cells (in a case of SCID patient)	No reactivity reported	Reactivity against CMV-infected cells like in the case of Vδ1 T cells	(57)
	Opsonization of CMV virions through CD16 (FcγRIIIa) expression and induction of IFN-γ responses	–	CD16 (FcγRIIIa) expression like Vδ1 T cells	(8)
HIV infection	Increased levels of Vδ1 T cells	–	–	(58)
	Co-expression of IFN-γ and IL-17 (defense against opportunistic infections), CD27 (memory phenotype), CCR7 (homing), and CD161 (transendothelial migration)	–	–	(59)
	Cytotoxicity against HIV-infected CD4^+^ T cells through NKG2C triggering	–	–	(60)
	Production of CCL3, CCL4, and CCL5 and suppression of HIV replication through NKp30 engagement	–	–	(61)
	–	Large production of IFN-γ, TNF-α, and the chemokines CCL4/CCL5: blockade of HIV co-receptors, attraction of more Vδ2^+^ T cells able to release additional chemokine blocking HIV entry and kill infected cells through direct cytotoxicity or ADCC	**–**	(62)
	–	Signaling through the CCR5-gp120 interaction: depletion of Vδ2^+^ T cells	–	(63)
Bacterial meningitis	–	Detection of IL-17^+^ Vγ9Vδ2^+^ lymphocytes in the peripheral blood and at the site of disease (a phenotype reversed by anti-bacterial therapy)	–	(14)
Mycobacterial infection	–	Control of mycobacteria replication through granzyme A and TNF-α (produced by macrophages)	–	(64)
Hepatitis C virus	Expansion and activation of Vδ1^+^ T cells in the liver Production of IFN-γ after polyclonal activation *in vitro*. Contribution to necroinflammatory liver disease because of their compartmentalization	–	–	(65, 66)
Human Herpes virus 8	Expanded Vδ1^+^ T cell populations with reactivity toward HHV8-infected cells *in vitro*	–	–	(67)

*ADCC, antibody-dependent cellular cytotoxicity; CMV, cytomegalovirus; EPCR, endothelial protein C receptor; SCID, severe combined immunodeficiency*.

During mycobacterial infection, human Vγ9Vδ2 T cells are potently able to inhibit intracellular mycobacteria growth through the secretion of granzyme A and TNF-α (64). In children with bacterial meningitis, an increased percentage of IL-17^+^ Vγ9Vδ2 lymphocytes was detected in the peripheral blood and infected tissues but could be reversed following anti-bacterial therapy (14).

Among Vδ2^−^ γδ T cells, different clones were demonstrated to be reactive against CMV-infected cells (55), in agreement with their expansion in the blood of CMV-infected patients (57, 68–70) (Table 2). More recently, the Vγ4Vδ5 γδ T-cell clone isolated from a CMV-infected transplant patient was shown to be reactive against CMV-infected endothelial cells as well as epithelial tumors after binding to the endothelial protein C receptor (56). In addition, CD16 expression by γδ T cells induces IFN-γ responses by opsonized CMV virions in a TCR-independent manner and, thus, contributes to the surveillance of CMV reactivation in transplant recipients (8). In a case of severe combined immunodeficiency patient, specific antibody responses to some infectious agents were reported with a predominance of Vδ2^−^ γδ T-cell clones reactive against CMV-infected cells, suggesting functional potentials of γδ T cells in providing B cell help (57). Therefore, γδ T cells do not only serve as sentinels in the innate system but can also act as a bridge between innate and adaptive immune responses.

Regarding the Vδ1 T-cell subset, their expansion and activation were also observed in many patients following viral infection such as HCV (hepatitis C virus) (65, 66) and HIV (58, 71). The specific depletion of Vδ2 T cells is the consequence of the virus infection through gp120-CCR5 interaction (63, 72). Nevertheless, uninfected Vγ9Vδ2 cells are able to produce large amounts of IFN-γ, TNF-α, and chemokines such as CCL4 and CCL5 [reviewed in Ref. (73)], which bind to CCR4 and CCR5 (the HIV co-receptors), respectively (74, 75). Interestingly, *in vitro* stimulation of Vγ9Vδ2 cells with phosphoantigens induces the release of these chemokines, saturates the co-receptors of HIV, and thus prevents HIV entry and interfere with its replication (76). It also permits the recruitment of more Vγ9Vδ2 cells able to release additional chemokines that would block HIV entry and kill infected cells through direct cytotoxicity or ADCC (62).

Freshly isolated Vδ1 T cells from HIV-infected patients were shown to express CD27, CCR7, and CD161, a molecule involved in γδ T-cell transendothelial migration and interestingly to proliferate and produce IFN-γ and IL-17 in response to *Candida albicans ex vivo* (59) (Table 2). These observations suggest that Vδ1 lymphocytes might play a major role in the control of HIV infection and in the defense against opportunistic infections. NKG2C was outlined as the major triggering receptor involved in the Vδ1 T-cell-mediated cytotoxicity against HIV-infected CD4^+^ T cells (60). The engagement of NKp30 on Vδ1 T cells induces CCL3, CCL4, and CCL5 production and suppress HIV replication (61). Altogether, these results are consistent with an antiviral potential of the Vδ1 T cells, possibly compensating the impairment of the CD4^+^ T-cell function during HIV infection.

Thus, the identification of stress-induced self antigens as targets expressed by infected cells may lead to the development of new therapeutic tools in infectious diseases. However, γδ T-cell-based therapy may give rise to an uncontrolled inflammation with unwanted tissue destruction. Therefore, exploiting the underlying mechanisms of γδ T-cell functions in infections will permit the modulation of their immunopathological consequences and could be beneficial to target pathogen proliferation.

### γδ T-Cell Subsets in Autoimmune and Other Chronic Inflammatory Diseases

γδ T cells were also reported to play a key role in various chronic inflammatory pathologies (Table 3). For instance, in a rare variant of myositis, referred to as γδ T-cell-mediated myositis, muscle fibers are attacked by CD8-negative T cells expressing the γδ-TCR. A Vγ1.3Vδ2 TCR clone (M88) was able to recognize the aminoacyl-histidyl-tRNA synthetase, an antigen also targeted by autoantibodies (77), suggesting a link between γδ T cells and antibody-dependent response in autoimmune myositis.

**Table 3:** Main features of human γδ T-cell subsets in chronic inflammatory manifestations.

Pathology	Human γδ T-cell subset features			Reference
	Vδ1^+^	Vδ2^+^	Other Vδ2^−^ (non-Vδ1)	
Myositis	–	Recognition of AA-RS (also targeted by anti-Jo-1 autoantibodies) by a specific clone of Vγ1.3Vδ2^−^ TCR (link between γδ T and B cells)	–	(77)
Psoriasis	–	Biomarkers for psoriasis, homing to the skin, production of pro-inflammatory cytokines (IFN-γ, TNF-α, and IL-17A), induction of immune cell recruitment from the circulation, release of growth factors and tissue remodeling	–	(78)
Rheumatoid arthritis	–	Antigen presentation capacity of effector-memory Vγ9Vδ2^+^ T cell stimulated with IPP to CD4^+^ T cells, secretion of pro-inflammatory cytokines (IFN-γ and IL-17), disease progression	–	(79)
Systemic lupus erythematosus	Decrease of CD27^+^CD25^high^FoxP3^+^ immunoregulatory Vδ1^+^ T cells	–	–	(80)

*AA-RS, aminoacyl-histidyl-tRNA synthetase; IPP, isopentenyl pyrophosphate*.

In rheumatoid arthritis, stimulation of effector-memory Vγ9Vδ2 T cell with isopentenyl pyrophosphate induces cell surface HLA-DR and co-stimulatory molecule expression and IFN-γ and IL-17 secretion. This consequently activated antigen presentation to CD4^+^ T cells, sustained T cell activation, and aggravated the disease (79).

A subset of CD27^+^CD25^high^ Vδ1 T cells, expressing FoxP3 similarly to regulatory CD4^+^ T cells, was reported in patients with systemic lupus erythematosus (SLE). Their immunoregulatory activity is mainly cell-to-cell contact dependent. Unfortunately, this population is decreased in the blood of SLE patients, which could be important for the understanding of the pathogenesis of this disease (80).

In psoriasis patients, a homing/redistribution of Vγ9Vδ2 T cells from blood to skin was observed. These cells produced an array of pro-inflammatory cytokines, such as IFN-γ, TNF-α, and IL-17A, which induced the recruitment of blood immune cells, the release of growth factors, and the tissue remodeling. A psoriasis-targeted therapy could normalize the decreased numbers of circulating Vγ9Vδ2 T cells indicating that they represent an important biomarker (78). Of note, skin-γδ T cells were reported to play an important role in wound healing (81).

Given that γδ T cells could influence the pathogenesis of chronic inflammatory diseases, these cells will require a balance between their need for inflammatory mediators to function normally and a negative regulation of their unfavorable impact of the chronic inflammation (82). Therefore, additional studies should be carried out on human γδ T-cell subsets to develop personalized γδ T-cell-based therapies.

## Conclusion and Perspectives

These studies give us more insights about the relative contribution of Vδ1 and Vδ2 T cells, as well as the mechanisms that could be targeted in order to develop efficient γδ T-cell-based therapies. However, more extensive investigation is required to better evaluate the impact of these cells, i.e., their effector function, the subset plasticity and cell lineage. Furthermore, a deeper characterization of γδ T-cell subsets in human is required given that most studies are conducted in mice. This will ultimately permit the manipulation of a specific subset according to a particular microenvironment. It is also important to understand how the novel therapies (e.g., ICP blockade) modulate γδ T-cell function. Finally, one may speculate that γδ T-cell subset-targeting could be beneficial to enhance or inhibit the outcome of adaptive immune responses against cancer, pathogens, or autoantigens, given their involvement in the regulation of other immune cell function.

## Author Contributions

ML wrote/revised the paper. JD-M and M-CD-N revised the paper.

## Conflict of Interest Statement

The authors declare that the research was conducted in the absence of any commercial or financial relationships that could be construed as a potential conflict of interest.
